# 1-[(6-Chloro­pyridin-3-yl)meth­yl]imidazolidin-2-iminium chloride

**DOI:** 10.1107/S1600536811053487

**Published:** 2011-12-17

**Authors:** Rajni Kant, Vivek K. Gupta, Kamini Kapoor, Madhukar B. Deshmukh, Chetan S. Shripanavar

**Affiliations:** a X-ray Crystallography Laboratory, Post-Graduate Department of Physics, University of Jammu, Jammu Tawi 180 006, India; b Department of Chemistry, Shivaji University, Kolhapur 416 004, India

## Abstract

The title compound, C_9_H_12_ClN_4_^+^·Cl^−^, is a natural metabolic product of imidacloprid [systematic name: (*E*)-1-(6-chloro-3-pyridyl­meth­yl)-*N*-nitro­imidazolidin-2-yl­idene­amine] and was obtained by the reduction of the latter using Fe in HCl. The dihedral angle between the pyridine and imidazole rings is 62.09 (12)°. The crystal structure is stabilized by N—H⋯Cl and C—H⋯Cl inter­actions involving the chloride anion. The pyridine N and the chloride atoms are not involved in inter­molecular inter­actions.

## Related literature

For background to the insecticidal applications of imidacloprid, see: Kanne *et al.* (2005 ▶); Schulz-Jander *et al.* (2002 ▶); Dai *et al.* (2010 ▶ **);** Tanner (2010 ▶). For ring conformations, see: Duax & Norton (1975 ▶). For related structures, see: Kapoor *et al.* (2011 ▶).
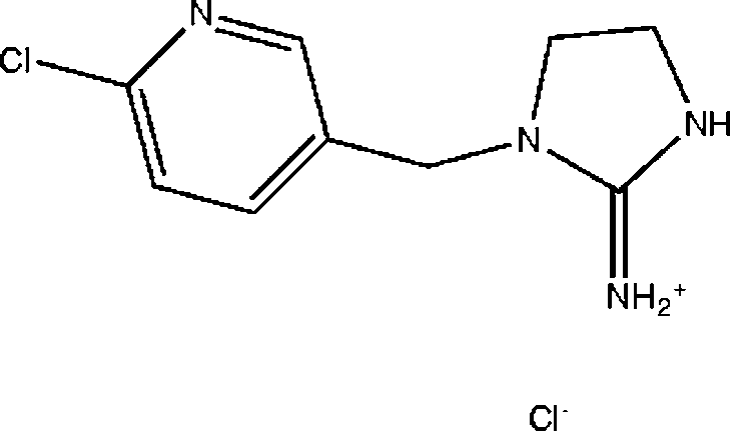


## Experimental

### 

#### Crystal data

C_9_H_12_ClN_4_^+^·Cl^−^
*M* *_r_* = 247.13
Triclinic, 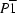

*a* = 6.4773 (3) Å
*b* = 7.3091 (3) Å
*c* = 12.4758 (4) Å
α = 88.996 (3)°
β = 77.214 (3)°
γ = 79.925 (3)°
*V* = 566.98 (4) Å^3^
*Z* = 2
Mo *K*α radiation
μ = 0.55 mm^−1^
*T* = 293 K
0.3 × 0.2 × 0.1 mm

#### Data collection

Oxford Diffraction Xcalibur Sapphire3 diffractometer
Absorption correction: multi-scan (*CrysAlis RED*; Oxford Diffraction, 2010 ▶) *T*_min_ = 0.742, *T*_max_ = 1.000
14139 measured reflections
2468 independent reflections
2059 reflections with *I* > 2σ(*I*)
*R*_int_ = 0.035

#### Refinement

*R*[*F* ^2^ > 2σ(*F* ^2^)] = 0.041
*wR*(*F* ^2^) = 0.108
*S* = 1.03
2468 reflections
137 parameters
H-atom parameters constrained
Δρ_max_ = 0.36 e Å^−3^
Δρ_min_ = −0.34 e Å^−3^

### 

Data collection: *CrysAlis PRO* (Oxford Diffraction, 2010 ▶); cell refinement: *CrysAlis PRO*; data reduction: *CrysAlis RED* (Oxford Diffraction, 2010 ▶); program(s) used to solve structure: *SHELXS97* (Sheldrick, 2008 ▶); program(s) used to refine structure: *SHELXL97* (Sheldrick, 2008 ▶); molecular graphics: *ORTEP-3* (Farrugia, 1997 ▶); software used to prepare material for publication: *PLATON* (Spek, 2009 ▶) and *PARST* (Nardelli, 1995 ▶).

## Supplementary Material

Crystal structure: contains datablock(s) I, global. DOI: 10.1107/S1600536811053487/gg2070sup1.cif

Structure factors: contains datablock(s) I. DOI: 10.1107/S1600536811053487/gg2070Isup2.hkl

Supplementary material file. DOI: 10.1107/S1600536811053487/gg2070Isup3.cml

Additional supplementary materials:  crystallographic information; 3D view; checkCIF report

## Figures and Tables

**Table 1:** Hydrogen-bond geometry (Å, °)

*D*—H⋯*A*	*D*—H	H⋯*A*	*D*⋯*A*	*D*—H⋯*A*
N13—H13*A*⋯Cl2	0.86	2.39	3.227 (2)	166
N13—H13*B*⋯Cl2^i^	0.86	2.33	3.177 (2)	169
N11—H11⋯Cl2^ii^	0.86	2.60	3.182 (2)	126
C7—H7*A*⋯Cl2^iii^	0.97	2.69	3.650 (2)	169
C7—H7*B*⋯Cl2	0.97	2.80	3.722 (2)	158

Symmetry codes: (i) 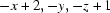; (ii) 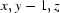; (iii) 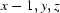.

**Fig. 1.**
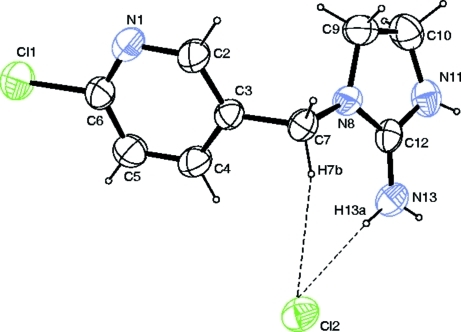
ORTEP view of the molecule with the atom-labeling scheme. The thermal ellipsoids are drawn at the 40% probability level. H atoms are shown as small spheres of arbitrary radii.

**Fig. 2.**
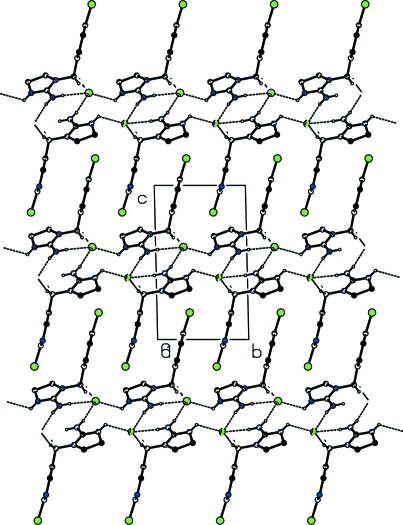
The packing arrangement of molecules viewed along the a axis. The dashed lines show the intermolecular N—H···Cl and C—H···Cl hydrogen bonds.

## supplementary crystallographic information

### Comment

Imidacloprid is one of the largest selling insecticides worldwide (Tanner, 2010). The discovery of imidacloprid has been referred to as a milestone in the past three decades of insecticidal research. Neonicotinoid insecticides act as antagonists on the pest synaptic nicotinic acetylcholine receptor (nAchRs) of the insect central nervous system (Tanner, 2010). The nitroguanidine moiety of imidacloprid is also a common site for metabolism *via* cleavage to the guanidine and reduction to des-nitro-imidacloprid. These metabolic modifications often result in an enhanced potency for vertebrate nAchRs and toxicity. (Kanne *et al.*, 2005; Schulz-Jander *et al.*, 2002; Dai *et al.*, 2010). The bond lengths and angles observed in (I) are normal and are comparable with related structures (Kapoor *et al.*, 2011). The imidazole ring adopts an envelope conformation with the asymmetric parameter: ΔCs(C10)=3.63 (Duax *et al.*, 1975). The dihedral angle between the C_5_N pyridine and C_3_N_2_ imidazole ring is 62.09 (12)°. The stabilization of crystal packing (Fig.2) is influenced by intermolecular N—H···Cl and C—H···Cl hydrogen bonds involving the chloride anion (Table 1).

### Experimental

Imidacloprid (12.75 g, 0.05 mol) was dissolved in 30 ml alcohol, fine powdered Fe (5.59 g, 0.10 mol) metal in the proportion of 1:2 was added, followed by 40 ml of conc. HCl. The mixture was refluxed for 10 hrs and the solid product was washed and cleaned by normal organic protocols, separated out, dissolved in alcohol and by the process of slow evaporation a yellowish crystalline compound was separated out. IR (KBr) νmax: 3233, 3083, 2923, 1690 cm^-1^. ^1^H NMR (300 MHz, CDCl_3_) δ: 3.55(s, 2 x CH_2_), 4.62(s, CH_2_), 7.36(d, J = 8.2 Hz, Py1H), 7.74(dd, J_1_= 7.5 Hz, J_2_ = 2.5 Hz, PyH), 8.21(s, NH), 8.32(s, Py1H) ppm. ^13^C NMR (300 MHz, CDCl_3_) δ: 159, 150, 149, 139, 130, 124, 100, 47, 45 ppm. LC—MS/MS (m/*z*): 211, 193, 175, 169, 133, 126, 84.

### Refinement

All H atoms were positioned geometrically and were treated as riding on their parent C/N atoms, with C—H distances of 0.93–0.97 Å; N—H distances of 0.86 Å and with *U*_iso_(H) = 1.2Ueq(C/N).

### Crystal data

**Table:**

C_9_H_12_ClN_4_^+^·Cl^−^	*Z* = 2
*M**_r_* = 247.13	*F*(000) = 256
Triclinic, *P*1	*D*_x_ = 1.448 Mg m^−^^3^
Hall symbol: -P 1	Mo *K*α radiation, λ = 0.71073 Å
*a* = 6.4773 (3) Å	Cell parameters from 7015 reflections
*b* = 7.3091 (3) Å	θ = 3.9–29.1°
*c* = 12.4758 (4) Å	µ = 0.55 mm^−^^1^
α = 88.996 (3)°	*T* = 293 K
β = 77.214 (3)°	Plate, yellow
γ = 79.925 (3)°	0.3 × 0.2 × 0.1 mm
*V* = 566.98 (4) Å^3^	

### Data collection

**Table:**

Oxford Diffraction Xcalibur Sapphire3 diffractometer	2468 independent reflections
Radiation source: fine-focus sealed tube	2059 reflections with *I* > 2σ(*I*)
graphite	*R*_int_ = 0.035
Detector resolution: 16.1049 pixels mm^-1^	θ_max_ = 27.0°, θ_min_ = 3.9°
ω scans	*h* = −8→8
Absorption correction: multi-scan (*CrysAlis RED*; Oxford Diffraction, 2010)	*k* = −9→9
*T*_min_ = 0.742, *T*_max_ = 1.000	*l* = −15→15
14139 measured reflections	

### Refinement

**Table:**

Refinement on *F*^2^	Secondary atom site location: difference Fourier map
Least-squares matrix: full	Hydrogen site location: inferred from neighbouring sites
*R*[*F*^2^ > 2σ(*F*^2^)] = 0.041	H-atom parameters constrained
*wR*(*F*^2^) = 0.108	*w* = 1/[σ^2^(*F*_o_^2^) + (0.0442*P*)^2^ + 0.3165*P*] where *P* = (*F*_o_^2^ + 2*F*_c_^2^)/3
*S* = 1.03	(Δ/σ)_max_ = 0.001
2468 reflections	Δρ_max_ = 0.36 e Å^−^^3^
137 parameters	Δρ_min_ = −0.34 e Å^−^^3^
0 restraints	Extinction correction: *SHELXL97* (Sheldrick, 2008), Fc^*^=kFc[1+0.001xFc^2^λ^3^/sin(2θ)]^-1/4^
Primary atom site location: structure-invariant direct methods	Extinction coefficient: 0.027 (4)

### Special details

**Table:**

Geometry. All e.s.d.'s (except the e.s.d. in the dihedral angle between two l.s. planes) are estimated using the full covariance matrix. The cell e.s.d.'s are taken into account individually in the estimation of e.s.d.'s in distances, angles and torsion angles; correlations between e.s.d.'s in cell parameters are only used when they are defined by crystal symmetry. An approximate (isotropic) treatment of cell e.s.d.'s is used for estimating e.s.d.'s involving l.s. planes.
Refinement. Refinement of *F*^2^ against ALL reflections. The weighted *R*-factor *wR* and goodness of fit *S* are based on *F*^2^, conventional *R*-factors *R* are based on *F*, with *F* set to zero for negative *F*^2^. The threshold expression of *F*^2^ > σ(*F*^2^) is used only for calculating *R*-factors(gt) *etc*. and is not relevant to the choice of reflections for refinement. *R*-factors based on *F*^2^ are statistically about twice as large as those based on *F*, and *R*- factors based on ALL data will be even larger.

### Fractional atomic coordinates and isotropic or equivalent isotropic displacement parameters (Å^2^)

**Table:**

	*x*	*y*	*z*	*U*_iso_*/*U*_eq_	
Cl2	0.86358 (8)	0.30462 (7)	0.60008 (4)	0.04939 (19)	
Cl1	0.23994 (12)	0.35819 (11)	1.17486 (5)	0.0720 (2)	
N1	0.1057 (3)	0.2611 (3)	1.00679 (16)	0.0649 (6)	
C2	0.1336 (3)	0.2148 (4)	0.89995 (19)	0.0587 (6)	
H2	0.0126	0.2007	0.8745	0.070*	
C3	0.3286 (3)	0.1868 (3)	0.82568 (15)	0.0365 (4)	
C4	0.5059 (3)	0.2069 (3)	0.86513 (18)	0.0500 (5)	
H4	0.6416	0.1874	0.8184	0.060*	
C5	0.4824 (4)	0.2558 (4)	0.97404 (19)	0.0544 (6)	
H5	0.6002	0.2700	1.0023	0.065*	
C6	0.2785 (4)	0.2826 (3)	1.03902 (16)	0.0473 (5)	
C7	0.3440 (3)	0.1424 (3)	0.70621 (15)	0.0380 (4)	
H7A	0.2113	0.1987	0.6860	0.046*	
H7B	0.4600	0.1959	0.6613	0.046*	
N8	0.3828 (3)	−0.0570 (2)	0.68359 (13)	0.0392 (4)	
C9	0.2100 (4)	−0.1652 (3)	0.7134 (2)	0.0510 (5)	
H9A	0.0927	−0.1189	0.6779	0.061*	
H9B	0.1550	−0.1632	0.7923	0.061*	
C10	0.3206 (4)	−0.3597 (3)	0.6710 (2)	0.0566 (6)	
H10A	0.3486	−0.4399	0.7308	0.068*	
H10B	0.2345	−0.4152	0.6305	0.068*	
N11	0.5199 (3)	−0.3268 (2)	0.59921 (15)	0.0503 (5)	
H11	0.6049	−0.4064	0.5525	0.060*	
C12	0.5523 (3)	−0.1553 (3)	0.61621 (15)	0.0388 (4)	
N13	0.7288 (3)	−0.0930 (3)	0.57049 (15)	0.0512 (5)	
H13A	0.7407	0.0194	0.5835	0.061*	
H13B	0.8325	−0.1646	0.5276	0.061*	

### Atomic displacement parameters (Å^2^)

**Table:**

	*U*^11^	*U*^22^	*U*^33^	*U*^12^	*U*^13^	*U*^23^
Cl2	0.0360 (3)	0.0522 (3)	0.0531 (3)	−0.0003 (2)	−0.0009 (2)	−0.0019 (2)
Cl1	0.0869 (5)	0.0993 (5)	0.0355 (3)	−0.0398 (4)	−0.0064 (3)	−0.0068 (3)
N1	0.0480 (11)	0.1002 (17)	0.0444 (11)	−0.0233 (11)	0.0042 (8)	−0.0210 (10)
C2	0.0351 (11)	0.0911 (18)	0.0490 (13)	−0.0136 (11)	−0.0034 (9)	−0.0215 (12)
C3	0.0359 (10)	0.0346 (9)	0.0362 (10)	−0.0034 (7)	−0.0042 (7)	−0.0012 (7)
C4	0.0356 (10)	0.0674 (14)	0.0445 (11)	−0.0099 (10)	−0.0025 (9)	−0.0040 (10)
C5	0.0448 (12)	0.0730 (15)	0.0499 (12)	−0.0173 (11)	−0.0145 (10)	−0.0023 (11)
C6	0.0590 (13)	0.0517 (12)	0.0330 (10)	−0.0199 (10)	−0.0059 (9)	−0.0001 (8)
C7	0.0378 (10)	0.0366 (10)	0.0361 (10)	−0.0015 (8)	−0.0045 (8)	−0.0021 (7)
N8	0.0363 (8)	0.0393 (9)	0.0390 (9)	−0.0055 (7)	−0.0023 (7)	−0.0044 (7)
C9	0.0455 (12)	0.0518 (12)	0.0554 (13)	−0.0158 (10)	−0.0048 (10)	0.0007 (10)
C10	0.0685 (15)	0.0456 (12)	0.0600 (14)	−0.0166 (11)	−0.0184 (12)	0.0011 (10)
N11	0.0540 (11)	0.0429 (10)	0.0514 (11)	0.0018 (8)	−0.0136 (8)	−0.0121 (8)
C12	0.0384 (10)	0.0437 (10)	0.0326 (9)	0.0016 (8)	−0.0106 (8)	−0.0033 (8)
N13	0.0386 (9)	0.0571 (11)	0.0497 (10)	−0.0015 (8)	0.0034 (8)	−0.0121 (8)

### Geometric parameters (Å, °)

**Table:**

Cl1—C6	1.743 (2)	N8—C12	1.328 (2)
N1—C6	1.305 (3)	N8—C9	1.460 (3)
N1—C2	1.346 (3)	C9—C10	1.522 (3)
C2—C3	1.376 (3)	C9—H9A	0.9700
C2—H2	0.9300	C9—H9B	0.9700
C3—C4	1.377 (3)	C10—N11	1.455 (3)
C3—C7	1.509 (3)	C10—H10A	0.9700
C4—C5	1.380 (3)	C10—H10B	0.9700
C4—H4	0.9300	N11—C12	1.334 (3)
C5—C6	1.372 (3)	N11—H11	0.8600
C5—H5	0.9300	C12—N13	1.313 (3)
C7—N8	1.457 (2)	N13—H13A	0.8600
C7—H7A	0.9700	N13—H13B	0.8600
C7—H7B	0.9700		
			
C6—N1—C2	115.96 (19)	C12—N8—C9	110.48 (17)
N1—C2—C3	124.6 (2)	C7—N8—C9	121.21 (16)
N1—C2—H2	117.7	N8—C9—C10	102.83 (18)
C3—C2—H2	117.7	N8—C9—H9A	111.2
C2—C3—C4	116.79 (19)	C10—C9—H9A	111.2
C2—C3—C7	121.09 (18)	N8—C9—H9B	111.2
C4—C3—C7	122.09 (17)	C10—C9—H9B	111.2
C3—C4—C5	120.0 (2)	H9A—C9—H9B	109.1
C3—C4—H4	120.0	N11—C10—C9	102.83 (18)
C5—C4—H4	120.0	N11—C10—H10A	111.2
C6—C5—C4	117.4 (2)	C9—C10—H10A	111.2
C6—C5—H5	121.3	N11—C10—H10B	111.2
C4—C5—H5	121.3	C9—C10—H10B	111.2
N1—C6—C5	125.2 (2)	H10A—C10—H10B	109.1
N1—C6—Cl1	115.99 (17)	C12—N11—C10	110.36 (17)
C5—C6—Cl1	118.74 (17)	C12—N11—H11	124.8
N8—C7—C3	112.18 (15)	C10—N11—H11	124.8
N8—C7—H7A	109.2	N13—C12—N8	125.00 (19)
C3—C7—H7A	109.2	N13—C12—N11	123.74 (18)
N8—C7—H7B	109.2	N8—C12—N11	111.26 (18)
C3—C7—H7B	109.2	C12—N13—H13A	120.0
H7A—C7—H7B	107.9	C12—N13—H13B	120.0
C12—N8—C7	126.77 (17)	H13A—N13—H13B	120.0
			
C6—N1—C2—C3	1.1 (4)	C3—C7—N8—C12	−118.6 (2)
N1—C2—C3—C4	0.5 (4)	C3—C7—N8—C9	76.9 (2)
N1—C2—C3—C7	−177.6 (2)	C12—N8—C9—C10	11.1 (2)
C2—C3—C4—C5	−1.0 (3)	C7—N8—C9—C10	177.87 (18)
C7—C3—C4—C5	177.1 (2)	N8—C9—C10—N11	−14.3 (2)
C3—C4—C5—C6	0.0 (4)	C9—C10—N11—C12	13.7 (2)
C2—N1—C6—C5	−2.3 (4)	C7—N8—C12—N13	10.4 (3)
C2—N1—C6—Cl1	175.7 (2)	C9—N8—C12—N13	176.29 (19)
C4—C5—C6—N1	1.8 (4)	C7—N8—C12—N11	−168.74 (18)
C4—C5—C6—Cl1	−176.16 (18)	C9—N8—C12—N11	−2.9 (2)
C2—C3—C7—N8	−91.8 (2)	C10—N11—C12—N13	173.4 (2)
C4—C3—C7—N8	90.2 (2)	C10—N11—C12—N8	−7.4 (2)

### Hydrogen-bond geometry (Å, °)

**Table:**

*D*—H···*A*	*D*—H	H···*A*	*D*···*A*	*D*—H···*A*
N13—H13A···Cl2	0.86	2.39	3.227 (2)	166
N13—H13B···Cl2^i^	0.86	2.33	3.177 (2)	169
N11—H11···Cl2^ii^	0.86	2.60	3.182 (2)	126
C7—H7A···Cl2^iii^	0.97	2.69	3.650 (2)	169
C7—H7B···Cl2	0.97	2.80	3.722 (2)	158

Symmetry codes:  (i) −*x*+2, −*y*, −*z*+1; (ii) *x*, *y*−1, *z*; (iii) *x*−1, *y*, *z*.

## References

[bb1] Dai, Y., Ji, W., Chen, T., Zhang, W., Liu, Z., Ge, F. & Yuan, S. (2010). *J. Agric. Food Chem.* **58**, 2419–2425. 10.1021/jf903787s 20112912

[bb2] Duax, W. L. & Norton, D. A. (1975). *Atlas of Steroid Structures*, Vol. 1. New York: Plenum Press.

[bb3] Farrugia, L. J. (1997). *J. Appl. Cryst.* **30**, 565.

[bb4] Kanne, D. B., Dick, R. A., Tomizawa, M. & Casida, J. E. (2005). *Chem. Res. Toxicol.* **18**, 1479–1484. 10.1021/tx050160u 16167841

[bb5] Kapoor, K., Gupta, V. K., Kant, R., Deshmukh, M. B. & Sripanavar, C. S. (2011). *X-ray Struct. Anal. Online*, **27**, x55–x56.

[bb6] Nardelli, M. (1995). *J. Appl. Cryst.* **28**, 659.

[bb7] Oxford Diffraction (2010). *CrysAlis RED* and *CrysAlis PRO* Oxford Diffraction Ltd, Yarnton, England.

[bb8] Schulz-Jander, D. A., Leimkuehler, W. M. & Casida, J. E. (2002). *Chem. Res. Toxicol.* **15**, 1158–1165. 10.1021/tx0200360 12230409

[bb9] Sheldrick, G. M. (2008). *Acta Cryst.* A**64**, 112–122. 10.1107/S0108767307043930 18156677

[bb10] Spek, A. L. (2009). *Acta Cryst.* D**65**, 148–155. 10.1107/S090744490804362X PMC2631630 19171970

[bb11] Tanner, G. (2010). MSc thesis, Austrian Agency for Health and Food Safety, Vienna.

